# Dr. Theron Augustus Wales

**Published:** 1914-10

**Authors:** 


Dr. Theron Augustus Wales, Pennsylvania, 1873, died at his home in Elmira, September 9, of chronic nephritis. He received his education at the Kimball Union Academy of Meriden, N. H., graduated in 1869, taught school for a time and studied medicine first at the University of Michigan, then at the University of Pennsylvania. He was connected with the Gleason Sanitarium for many years but, after 1897, was in private practice in Elmira. He was a prominent mason, had been one of the police commissioners of Elmira and had held various other positions of honor. He had been President of the Chemung County Medical Society and of the Third District Branch of the State Society. His wife, Dr. Zippie Brooks Wales died July 4, 1912.

